# Challenges and recommendations for wearable devices in digital health: Data quality, interoperability, health equity, fairness

**DOI:** 10.1371/journal.pdig.0000104

**Published:** 2022-10-13

**Authors:** Stefano Canali, Viola Schiaffonati, Andrea Aliverti

**Affiliations:** Department of Electronics, Information and Bioengineering, Politecnico di Milano, Milan, Italy; Vanderbilt University, UNITED STATES

## Abstract

Wearable devices are increasingly present in the health context, as tools for biomedical research and clinical care. In this context, wearables are considered key tools for a more digital, personalised, preventive medicine. At the same time, wearables have also been associated with issues and risks, such as those connected to privacy and data sharing. Yet, discussions in the literature have mostly focused on either technical or ethical considerations, framing these as largely separate areas of discussion, and the contribution of wearables to the collection, development, application of biomedical knowledge has only partially been discussed. To fill in these gaps, in this article we provide an epistemic (knowledge-related) overview of the main functions of wearable technology for health: monitoring, screening, detection, and prediction. On this basis, we identify 4 areas of concern in the application of wearables for these functions: data quality, balanced estimations, health equity, and fairness. To move the field forward in an effective and beneficial direction, we present recommendations for the 4 areas: local standards of quality, interoperability, access, and representativity.

## Introduction

Devices that can be worn on our bodies and track several activities and parameters—*wearable devices*—are increasingly sold and used in the general population. One of the main areas of use of wearable devices is health, including biomedical research, clinical care, personal health practices and tracking, technology development, and engineering. In this context, the use of wearables for health has been connected to several promises and benefits for a more digital, personalised, preventive medicine [1–3]. At the same time, crucial work has identified and discussed technical and ethical challenges in the extended use of wearables for health, including accuracy, privacy, security, cyber risks [4–7]. Yet, most analyses have focused on either of these areas of discussions, thus framing technical and ethical considerations as largely separate issues. As a result, the connections between specific technical solutions and ethical considerations remain underdiscussed: This is a problem, as we will show that many challenges of the wearable context can be addressed only partially through technical solutions. In addition, the epistemic (knowledge-related) contribution of wearables to the collection, development, application of biomedical knowledge has only partially been discussed. As a result, there is a lack of understanding of the specific uses and functions that wearables can and should fulfil for digital health—yet this is crucial to identify the role of wearables in digital health and beyond and assess their ethical and social impact in relation to specific uses.

In response to these considerations, in this article we start by providing an epistemic overview of the main functions of wearable technology for health: we discuss *monitoring*, *screening*, *detection*, and *prediction*. The role of these functions is clear when looking at the use of wearables in concrete cases (Table 1), for example, the context of Coronavirus Disease 2019 (COVID-19). The COVID-19 pandemic has been discussed as a crucial catalyst for the use of wearable technologies in the biomedical and health domain and we will use it as a source of uses and cases to illustrate our points throughout the article [8,9]. On the basis of this overview, we discuss specific issues and concerns that are connected to the use of wearables for the identified functions of monitoring, screening, detection, and prediction. We focus on 4 main areas of concern (data quality, balanced estimations, health equity, and fairness) and propose recommendations and possible solutions (local standards of quality, interoperability, access, and representativity). On the basis of our overview and analysis of these challenges, the recommendations we propose enable us to better understand the actual impact, benefits, and risks of wearables and improve their application for digital health (Table 2). In this way, as a group of researchers with different areas of expertise (biomedical engineering and research, philosophy and ethics of science and technology) but working in the same department, we develop an interdisciplinary account of wearable technology and its contribution to digital health.

**Table 1:** The main functions served by wearables for health, with examples.

Functions	Examples
Monitoring	- Pulse monitoring [10] - Advanced tele-monitoring [20] - COVID-19 symptoms and long-term effects monitoring [21]
Screening	- Atrial fibrillation screening [14] - Sleep apnea screening [13] - Cardiovascular disease screening [22]
Detection	- Physical activity levels detection [23] - Pre-symptomatic detection of COVID-19 infections [17] - Seasonal influenza detection [24]
Prediction	- Prediction of mortality and clinical risk [16] - Prediction of COVID-19 infections [17] - Prediction of exacerbations of chronic obstructive pulmonary disease [19]

**Table 2:** Summary of the identified areas of concern and key issues and proposed recommendations.

Areas of concern	Key issues	Recommendations	References
Data quality	- Variability of sensors, data collection practices - Lack of contextual information	Local standards of data quality	[27,29,34]
Balanced estimations	- Overestimation - Overprediction	Interoperability of wearable data	[35,41,44]
Health equity	- Unequal access to benefits - Digital and technological divides	Access to wearable data and interpretation	[28,50,52]
Fairness	- Exclusion of portions of the general population - Unfair wearable datasets	Representativity of wearable data	[2,9,36]

### An overview of wearable technology for digital health

There is currently an abundance of uses of wearable devices for health. Focusing on the epistemic contribution of wearables to the collection, development, application of biomedical knowledge, we develop an overview that looks at the current context of wearable technology. While this is not a systematic representation of all possible or future applications of wearable devices, we identify 4 main functions that wearables are currently used to serve in the health context: monitoring, screening, detection, and prediction (Table 1).

We identify *monitoring* as the basic and fundamental function served by wearables, performed by wristbands, patches, watches, clothing. Monitoring is the practice of continuous data collection, focused on members of a population, which can be the general population or a specific subset of individuals. Wearables are considered particularly efficient to fulfil this function because they can track a number of various biomedical processes depending on the types of sensors available and can be used for continuous and remote monitoring—as wearables can be worn constantly, they are ideally placed to collect data continuously. In this way, wearables can deliver a significant improvement to remote and tele-monitoring [10,11] and have been used in this sense to monitor crucial physiological metrics for COVID-19 such as heart rate, physical activity, oxygen saturation, as well as long-term effects [8,12]. In this context, wearable devices have also been applied in coordination with other tele-health systems for remote monitoring for individuals at risk that could easily shift to hospitalisation and to assist remote diagnosis.

On the basis of these monitoring capabilities, we identify 3 other main functions that wearables can serve. *Screening* is the identification of specific conditions and individuals associated with this condition within datasets collected through monitoring. The use of wearables for this function is usually based on passive sensors that measure motion, steps, light, pressure, sound, etc. [3]. For example, wearable garments have been used to monitor individuals during sleep and screen for individuals suffering from sleep apnea [13]. A close function related to screening is *detection*. When wearables monitor specific conditions in populations, they are often used to detect conditions and alert individual users. Detection is the analysis of wearable data collected through monitoring in order to investigate possible patterns and features that can be interpreted as indicators and markers of specific biomedical conditions. For example, a combination of smartwatches and dedicated bands has been used for heart rate monitoring and automatic detection of atrial fibrillation [14]. The integration of wearable data with symptom data has been presented as a way to improve the identification of COVID-19 positive versus negative cases [15]. Detection is also the function where we see an intersection with both monitoring and screening: for example, smartwatches have been used to monitor populations for irregular pulse and, on this basis, screen for individuals potentially suffering from atrial fibrillation as well as identify the condition [10]. A final diagnosis of a condition can thus be based on detections performed by wearables, although wearables currently cannot perform diagnosis as a consequence to technical and regulatory limitations.

The fourth function we identify is *prediction*, the inference of future trends and/or events of interest for the biomedical study of populations based on monitoring. Just a few wearable devices are currently used for prediction in the health context, for example, to predict mortality, readmissions, and clinical risk [16]. In the context of COVID-19, wearables have been tested for the retrospective detection of infection and prediction of COVID-19, days before the presence of symptoms [17]. Other examples include the use of accelerometer data from wearable devices to predict biological age and mortality [18] and respiratory rate data to predict exacerbations of chronic obstructive pulmonary disease [19].

These 4 functions are often intertwined and interconnected in concrete contexts and in many cases the same device can perform more than 1 function. Still, identifying different functions is a crucial step to understand the actual impact and goals of using wearable technology for health. Depending on whether we use wearables to predict or monitor health, different assumptions, uses, and standards will be necessary. In addition, understanding which functions wearables can and do serve currently helps us to make sense of their possible limitations. As we will see in the next sections, this overview enables us to see how the use of wearables for health is currently limited by crucial challenges, which impact different functions in different ways. The remainder of the paper will be dedicated to a discussion of these challenges, as they emerge in concrete uses of wearables to serve the functions we have identified.

### Local standards for data quality

In our overview, we have identified monitoring as the fundamental feature at the basis of the functions of wearable technology for health. Monitoring is a promising application of wearables thanks to their abilities for constant and personal data collection, but a key concern is *data quality*. Quality is a crucial feature of scientific data, which needs to be evaluated to warrant the reliability of scientific claims. Data quality is also one of the fundamental values of research ethics and the social goals of biomedical research—high-quality data are considered the basis for benefits at the clinical level and beyond [25]. Yet, the variability of sensors and lack of consistency of data collection in the wearable context make it difficult to coordinate and assess quality. In addition, the lack of contextual information on the ways in which wearable data are collected, classified, and interpreted raise concerns on the possibility of assessing quality.

A first issue that makes it difficult to assess data quality in the wearable context is variability. Wearable data are usually collected by different types of devices or different sensors, if not through different data collection practices. For example, the measurement of metrics such as oxygen saturation can vary substantially in terms of location (e.g., wrist, finger, ear) and types of devices (e.g., watches, rings, earphones) employed for measurement [1,11]. This level of variability makes it difficult to have common standards to assess data quality: The same parameter is often measured with very different sensors, which employ different processing techniques, and may even render different results [3]. One way of responding to these concerns is regulation, which should make sure that wearables can be used as reliable and high-quality sources of data. In this context, the push is to regulate wearables as medical devices on the basis of clinical validity [26]. Clinical validity is a crucial step for the adoption of wearable technologies for health and also for the regulation of the quality of wearable data.

Yet, clinical validity as an intrinsic feature of quality is not enough on its own. Extensive work in philosophy of science has shown that quality is not only an intrinsic feature of data. Quality is a contextual component of data: depending on a specific use and context of use, considerations of data quality might change [27]. For example, it is clearly crucial to know that a wearable device has been clinically validated to collect high-quality data on heart rate [22]. However, using a wearable to monitor heart rate and detect COVID-19 infection on this basis constitutes a new context of use, where considerations of data quality may be different. For example, a certain number of false positives might be considered good enough for fitness tracking or even remote monitoring of heart patients, but it might not be enough when wearables are used to detect COVID-19 and suggest isolation and quarantines. The ethical and social burdens of poor quality or unreliable data change depending on the context. For patients who rely on wearables to track the health of their heart after bypass surgery, the quality of data is more serious than for users tracking fitness activities: In the context of heart patients, the collection of low-quality data about severe health problems can be a very serious burden, leading to unnecessary anxiety [28]. For users with limited financial resources, wearables can be an inexpensive tool to keep track of their health when other health services are too expensive and difficult to access [2]. As such, wearables can improve access to healthcare, but wearable technology might constitute the main health service available for these users and poor data quality will unequally impact them more than others. This is why data quality should be considered a contextual property of data that needs to be constantly considered as the context of use changes significantly.

In turn, in order to assess data quality for a specific use, knowledge of contextual features of data collection is crucial. For example, it is crucial to know which experimental procedures and protocols were applied, which sensors and techniques were used, and which questions and hypotheses were investigated during data collection. As questions and hypotheses change from general heart monitoring to COVID-19 detection, for example, it is crucial to know the original experimental procedures and questions to understand whether data quality remains the same—access to contextual features of data practices is crucial to assess quality and ensure the reliability and trustworthiness of data [29]. The problem is that access to these contextual features is often not available in the wearable context. For example, the collection of heart rate data from wearable devices is usually covered by the opt-in of users to general medical studies, which are organised by private companies and large research bodies, such as the Apple Heart Study created by the collaboration between Stanford University and Apple. While this type of study was of course validated and regulated as a clinical trial [30], there is little information on data collection, analysis, storing, and access and this makes it difficult to assess quality. In addition, in many cases biomedical researchers cannot even download data directly from the device and have to go through proprietary archives. As a result, because of commercial interests, very little information on how the data are collected, classified, and interpreted by the device is shared throughout the process. This is an issue for researchers, but also users and patients. The lack of access to contextual information about data collection makes it difficult for users to interpret the data to take action on their health and can eventually lead them not to trust and use the technology [28,31,32].

On this basis, we need more contextual information and coherence for wearable data quality [33]. Contextual information can be used to understand the specific features and needs for the assessment of data quality in the wearable context. In this direction, common and local standards of data quality can be developed to overcome current limitations and gaps in the wearable market. For example, the framework provided by FAIR (**F**indability, **A**ccessibility, **I**nteroperability, and **R**euse) can be used as a basis to discuss future developments in this direction [34–36]. We do not see these as hard compliance standards set by standard organisations, but rather the result of a bottom-up process of coordination and assessment, as a way of fully appreciating the contextual dimensions of data quality. As we have seen, depending on the specific context of use, standards, requirements, and burdens of data quality can change. This is why data quality standards need to be local and could first be created for the research context, where knowledge of the contextual components of data collection is crucial to assess data quality. Yet, standards of data quality can clearly be crucial for regulators, institutions, industry, and users too, and standards could be adopted by the institutions, journals, repositories of specific research communities. Again similarly to FAIR data, the institutions of specific research communities could be in charge of managing, updating, and assessing standards and informing individual users of their existence and application, thus presenting data quality as a fundamental issue for digital health.

### Interoperability for balanced estimations

As we have seen in our overview, detection and prediction are among the main functions for which wearable devices are currently used in health. In turn, detection and prediction are fundamental activities at the basis of the production and use of scientific knowledge. Yet, issues affecting *balanced estimations* in screening and prediction raise concerns on the grounding and validity of wearables as detection and prediction tools.

In the COVID-19 pandemic, several models have been used to predict the development, spreading, and impact of the pandemic, but they have also been at the centre of several critiques concerning their uncertain assumptions and limitations [37–40]. Wearable devices have been proposed as potential solutions to some of these issues [8]. For example, data from Fitbits have been used to detect elevated signals at the level of heart rate and temperature—these are possible symptoms of COVID-19 that can be identified in advance or just when more explicit symptoms surfaced [17]. This is an extremely promising use of wearables, but the status of predictions based on wearable data raises challenges. Applications of wearables for the detection of COVID-19 are severely affected by overestimation, the issue where non-problematic conditions and abnormalities are systematically detected or predicted as problematic. For example, it is often difficult to differentiate between COVID-19 and seasonal influenza and cases of standard influenza on the basis of wearable data—elevated heart rate can be interpreted as a symptom of respiratory illness more generally and, as a result, wearables have wrongly detected and predicted COVID-19 infections [17,24].

This is a crucial epistemic issue for testing the validity of using wearable data to perform or assist prediction, but is also significant from an ethical point of view. For example, health resources and personnel may be diverted from actually problematic situations towards overestimated issues, thus creating imbalance in health treatment and access [41]. Erroneous prediction and detection can also create unnecessary stress in patients, raising concerns on the implementation of wearables for health [7,42]. In addition, the burdens of overestimation may also be unequally distributed over different types of social groups, policy contexts, healthcare services. For example, estimation issues in the context of COVID-19 might be overcome with access to molecular or antigen tests, which can differentiate between influenza and COVID-19. In this sense, it could be argued that overestimating infections might thus be better in light of the precautionary principle. However, access to fast COVID-19 testing is not equally available and distributed in the world and has often become expansive, especially when infections surge. Policy decisions might require a person to isolate if their wearable device has detected a possible infection (as we have seen with the use of contact-tracing smartphone apps), which is potentially harmful for them and their family, especially if remote work is not an option and wages might be lost. These issues are even more severe in the context of wearables and other digital health solutions. These technologies are presented as key opportunities for parts of the world with limited or non-existing health services [43]. However, if other technologies and services that might help overcome overestimation are limited and not available (e.g., fast COVID-19 testing), this poses even more significant constraints on the accuracy and estimation of wearables and other digital health technologies. Unsurprisingly, recent work by political institutions, such as the EU Commission, on the internet of things technologies such as wearables has concluded that overestimation is among the main issues for the adoption of wearable technology [2].

In order to overcome these concerns, we propose to focus on the interoperability of wearable data as a crucial way forward. Interoperability is the possibility that data can be integrated and used together with other types of data [35]. Several philosophical, historical, and sociological studies of the role of data in science have highlighted that the value of large volumes of data for research lies in the possibility of integrating and linking different datasets [44]. In this sense, a high level of interoperability is key to exploit the benefits of new and large datasets, such as those collected with wearable technology. A low level of interoperability makes it difficult to integrate wearable data with other health data and thus compare and balance results collected by different devices, sensors, approaches. In turn, making sure that wearable data are interoperable can make it easier to compare results obtained through other means and assess the extent to which overestimation might be a problem. Data interoperability is also connected to interoperability at the software and hardware level of wearable technology. For example, the integration of wearables into health services is currently challenging because the additional staff required to assist patients with the technology might need to be trained differently, as software and hardware solutions are different between devices [45]. In turn, interoperability standards are also crucial for data storage and thus to include wearables in health services, for example through personal and electronic health records, which is currently very costly [3], and to deal with cyber risks, for example by highlighting transparency and accountability in healthcare infrastructures [46,47]. Ensuring that a wearable device is interoperable is thus an essential way to approach overestimation and the promise of providing more personal and precise healthcare in digital health.

### Access for health equity

One of the defining features of wearables is their ability to be worn on our bodies. This means that they can often be personal devices, in the sense that they might fulfil a personal need of the user (such as tracking fitness activities and exercise) as well as be used as personal and fashion objects (such as rings and wristwatches). As such, wearables play a crucial role towards an increasingly personalised, precise, and person-centred medicine. In this way, wearable technology is uniquely positioned to move in the direction of one of the goals of digital health: expanding access to health services and thus improving *health equity*. Health equity is about making sure that different users are equally provided with services and care as part of their interactions with the health system, as defined in several policy initiatives such as the Thirteenth General Programme of Work of the World Health Organization (WHO). While we agree the contribution of wearables to these goals is promising, issues connected to access raise significant concerns.

As we have seen, wearable devices can clearly provide data that are personal to user needs, issues, and concerns [48]. However, the extent to which individual users can access the benefits of this data collection seems unequally distributed. Users with more digital literacy and socioeconomic resources are disproportionately advantaged to access benefits from the use of wearables as tools to detect and predict states of health and disease [49,50]. In addition, the use of wearables and other digital health tools for monitoring in the context of public health efforts might raise concerns about surveillance, in different ways for different social groups. Historically, members of marginalised social groups have been targeted by health surveillance and monitoring with unclear benefits and sometimes harmful results. For example, COVID-19 surveillance and policy restrictions have disproportionately affected structurally disadvantaged social groups [51]. If wearables as digital health technologies are to be made part of public health policy and campaigns, access to the technology needs to be ensured as much as access to clear benefits from the use of the technology. Currently, the benefits of health monitoring through wearables are disproportionately available to consumer technology companies, rather than individual users. Most wearables available on the market are developed and sold by some of the largest corporations in the world, such as Apple and Google. The increasing collection of health data through wearables by consumer technology creates clear economic and political benefits for these corporations, which can use the data for marketing and advertising. Individual users do not necessarily have access to these benefits of data collection or at least not at the same level [52].

In addition, even for those who can and do use wearables, other issues of access raise concerns on health equity. As we have seen, contextual information on the collection, classification, interpretation of wearable data is usually not shared by data providers and device manufacturers. This is an issue for health equity: epistemically, information of the ways in which wearable data are analysed for detection and screening is crucial to interpret data and translate results into significant actions of health promotion for individual users. Without this information, users can struggle to understand why the analysis of wearable data leads to the detection of a condition and how they can act upon this function. This can also create doubt and anxiety, as users do not know the extent to which the data are reliable and are unsure about the actions they can take to counter possibly alarming conclusions [28]. In other words, this creates a situation of health inequity. For some users, the collection of wearable data can be a source of actions to improve their health, but for others barriers to data access can create new burdens.

Several approaches have been proposed in recent years to counteract the burdens of health inequity [53]. A way forward for these challenges in the wearable context is an expansion of both the access to the data and related interpretation tools. More access to data can partially counter the economic and political power of technology corporations [54]. Access to interpretation, in turn, can empower users, enabling them to make sense of the trustworthiness, quality, and actionability of the functions provided by wearables. We see these as goals that should be part of health campaigns and public health policy involving wearables and other digital health technologies. Crucially for health equity, however, access to technology should be approached critically, in light of considerations of the specific social and political context of use. For example, some members of the general population might not be interested in tracking their health or might find it confusing, alienating, guilt-inducing, stressful. The specific use and position of wearables as digital health technology needs to be openly and critically discussed to ensure that those who choose not to be part of the movement are not unequally treated and loose access to other health services.

### Representativity for fairness

Wearables are at the centre of several attempts to make health more mobile and digital. As we have seen in our overview, wearables are technologies that can track and collect digital data on various daily activities and provide users with individual monitoring and screening in connection to other digital tools and services. Wearables can also be ways of further developing remote detection and prediction, without the need to interact with other health services. In the digital health context, this use of digital devices and services such as wearables is connected to various benefits. For example, digital health is often framed explicitly as an opportunity to shift the medical knowledge system towards the representation of the majority that is typically excluded from more traditional research methodology [55]. While wearables are clearly promising tools to achieve these crucial goals, we raise concerns on their *fairness*. In the health context, fairness is close to the notion of equity and related attempts for the equal distribution of services and care. Yet, fairness is also about the just treatment of individuals when they interact with health services and thus about making sure that people are not treated in unjust ways in healthcare because of bias, discrimination, lack of consideration [56]. We argue that current use and features of wearables disproportionately target some members of the general population and exclude others, thus creating issues of fairness.

Thanks to wearable and other digital devices, data points such as steps have been tracked for almost a decade, at a scale that is unprecedented when compared to more traditional and preceding data practices. In these ways, more generally, wearables are contributing to the increasing datafication of activities and aspects of our lives. But they are contributing to their medicalisation too, as the possibility of quantifying and measuring these activities and aspects renders them as new areas of research and intervention. In the health context, current processes of datafication and medicalisation are contributing to a re-configuration of health, by expanding the limits and remits of biomedical research, producing new markers of health and disease, redefining what counts as health data, broadening the categories of influential stakeholders, and involving and empowering more individuals [32]. Datafication and medicalisation through wearables can thus create various benefits by uncovering new health needs and issues of specific communities. Consider, for example, the role that patient groups have played throughout the COVID-19 pandemic in raising concerns on the limitations and diverse impact of public health interventions and raising awareness on the long-lasting effects of COVID-19 infection, which are now known as long COVID. Enabling patients to track their own health individually and actively can provide them with more powerful tools and empowerment in this direction.

However, current uses and applications of wearable technology for health focus only on some members and groups of the general population, thus rendering the use of the technology unfair. For example, consider the framing of wearable technology as a crucial tool for the remote and constant monitoring of the elderly and patients that need to practice social distancing, avoid hospital visits but require monitoring [8]. Looking at current figures on the adoption of wearable devices, members of the population that fit into these categories are severely underrepresented and excluded by the application of this technology [2]. This is highly problematic from the point of view of fairness: Wearable technology seems to exclude the users that arguably would benefit the most from the use of wearables. Children are also an interesting type of users in this sense. Age groups including young adolescents and children have increasing access to digital technologies, including wearables. Yet, the adoption of wearable technology in children can vary substantially, for example depending on whether they use other technologies (e.g., smartphones are normally gateways for wearables), where they live, and the socioeconomic status of their family. The new contribution of these age groups to biomedical research is an exciting opportunity of wearable technology, potentially enabling the retooling of medical knowledge system to represent groups that are currently excluded and underrepresented [55]. At the same time, the opportunity of further introducing wearable technology in these age groups needs to be balanced against ethical reflections about security, privacy, intrusiveness. More generally, the cases of the elderly and children suggest that, however, large and extended wearable datasets may be, wearables usually target some social, economic, age groups more than others. This is crucial because excluding important and large parts of the general population can lead to biased and underrepresentative datasets, which do not give us a good picture of population health, thus creating a weak and unsound basis for knowledge claims and focusing health policy only on few members of the population.

Thus, issues of fairness raise concerns on the legitimacy of using and recommending wearable technology for health in the general population. To overcome these challenges, more focus needs to be given on the representativity of various members of the general population in wearable technology and digital health. We see the focus on representativity as one of the steps of the assessment of data quality and fairness [36], which should be one of the first steps for discussions on using wearables as part of health promotion and public health programmes too. In addition, focusing on representativity can also be a way of taking into account the context around the use and introduction of wearable technology. In communities and parts of the world with limited availability of fast and inexpensive testing, for example, early detection of pre-symptomatic COVID-19 is not as useful or might be useful only for some members of the population, thus creating issues of fairness. Consider one of the prime areas of application of wearables for health: the tracking of physical activity to suggest interventions and behavioural change [57,58]. Wearables can be powerful tools in this context—yet alerting a person that they have been sedentary might not be as useful, if they do not have opportunities or services that can make them more active.

## Conclusions

In this paper, we have discussed various implications of wearable technology for digital health. First, we have identified functions that wearable technology currently serves in biomedical research and clinical care as a way of specifying the epistemic contribution of wearables to the development and application of biomedical knowledge through monitoring, screening, detection, and prediction (Table 1). On this basis, we have discussed a number of challenges that are connected to these functions, particularly at the level of data quality, estimations, equity, and fairness. As a way to overcome these challenges, we have introduced recommendations and possible solutions based on local standards of quality, interoperability, access, and representativity (Table 2). Our analysis has thus been aimed at improving our understanding of the position and relations between wearables and other biomedical technologies and data sources, as well as ways to approach their adoption and regulation.

Throughout the article, we have applied an integrated approach for the discussion of wearables for health, which we see as a starting point for more work. In recent years, philosophers, sociologists, and ethicists of science and technology have started to work more closely in collaboration with biomedical scientists, engineers, and practitioners. An increasing number of publications is the result of collaborations between science scholars and scientists; philosophical work is increasingly relevant and cited in science journals [59]. In this context, approaches such as ELSI (Ethical, Legal and Social Issues) and E^2^LSI show the need for a systematic integration of epistemic, ethical, legal, and social considerations [60]. This is a particularly important step to take in the context of new and evolving technologies for digital health, as important decisions are being taken now on their regulation, inclusion in healthcare programmes, and use in research. Our work in this article provides a first step for thinking about these as integrated issues.
